# Supplementary figures and images for: Genome-wide association study to identify the genomic loci associated with wheat heading date variation under autumn-sowing conditions
Source: PLoS One. 2025 Apr 30;20(4):e0322306. doi: 10.1371/journal.pone.0322306 (PMC12043121; doi:10.1371/journal.pone.0322306)

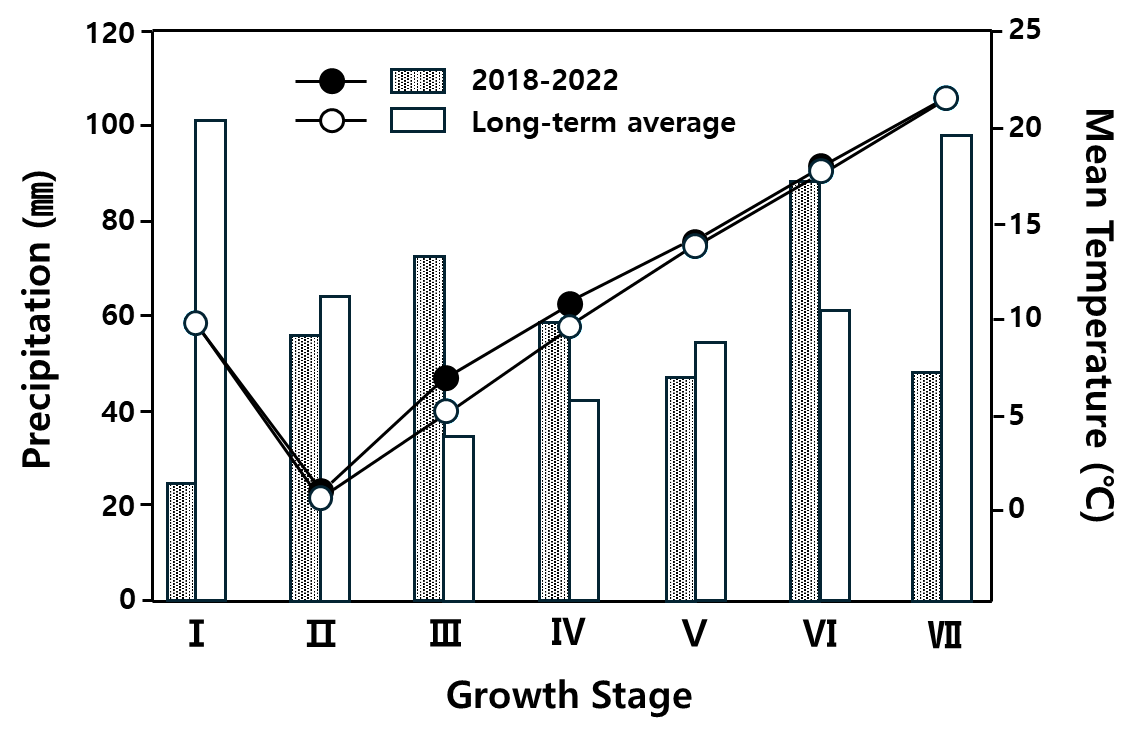

Supplement: S1 Fig — I, II, III, IV, V, VI, and VII represent the emergence, regeneration, tillering, elongation, heading, milky, and maturation stages, respectively. (TIF) [file pone.0322306.s001.tif]

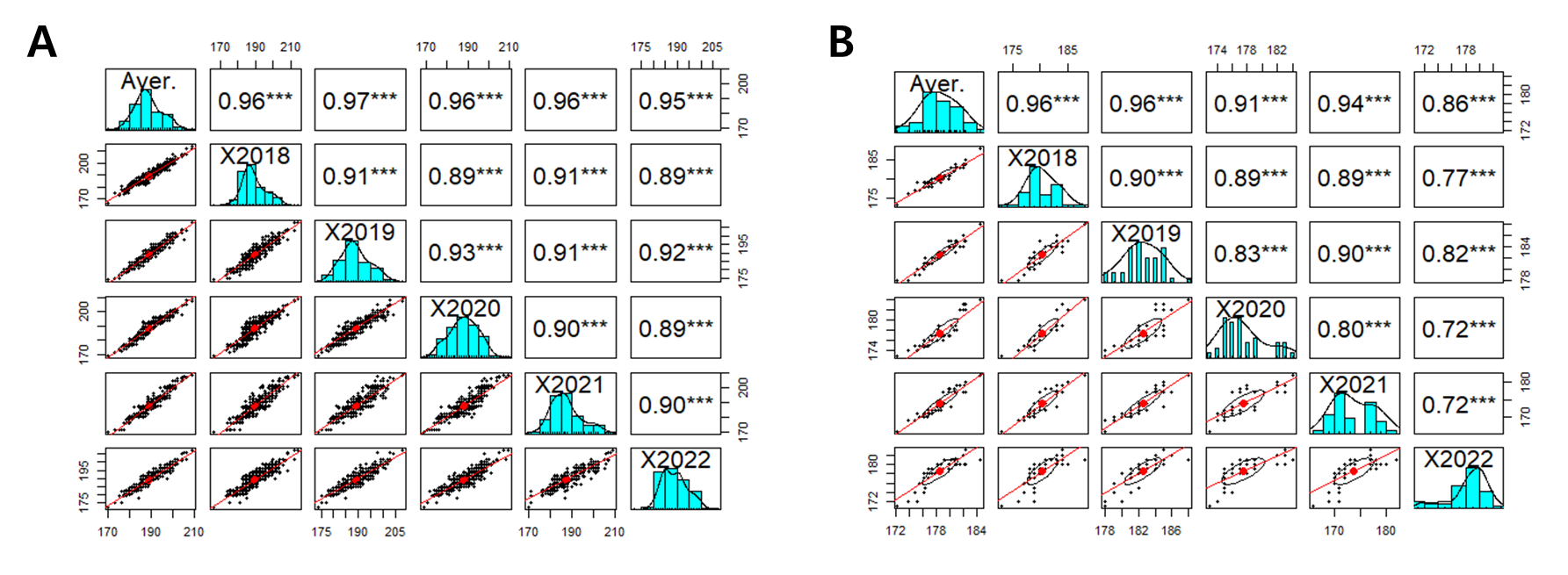

Supplement: S2 Fig — (A) Wheat core collections (n = 530). (B) Korean wheat varieties (n = 40). *** significant at P < 0.001. (TIF) [file pone.0322306.s002.tif]

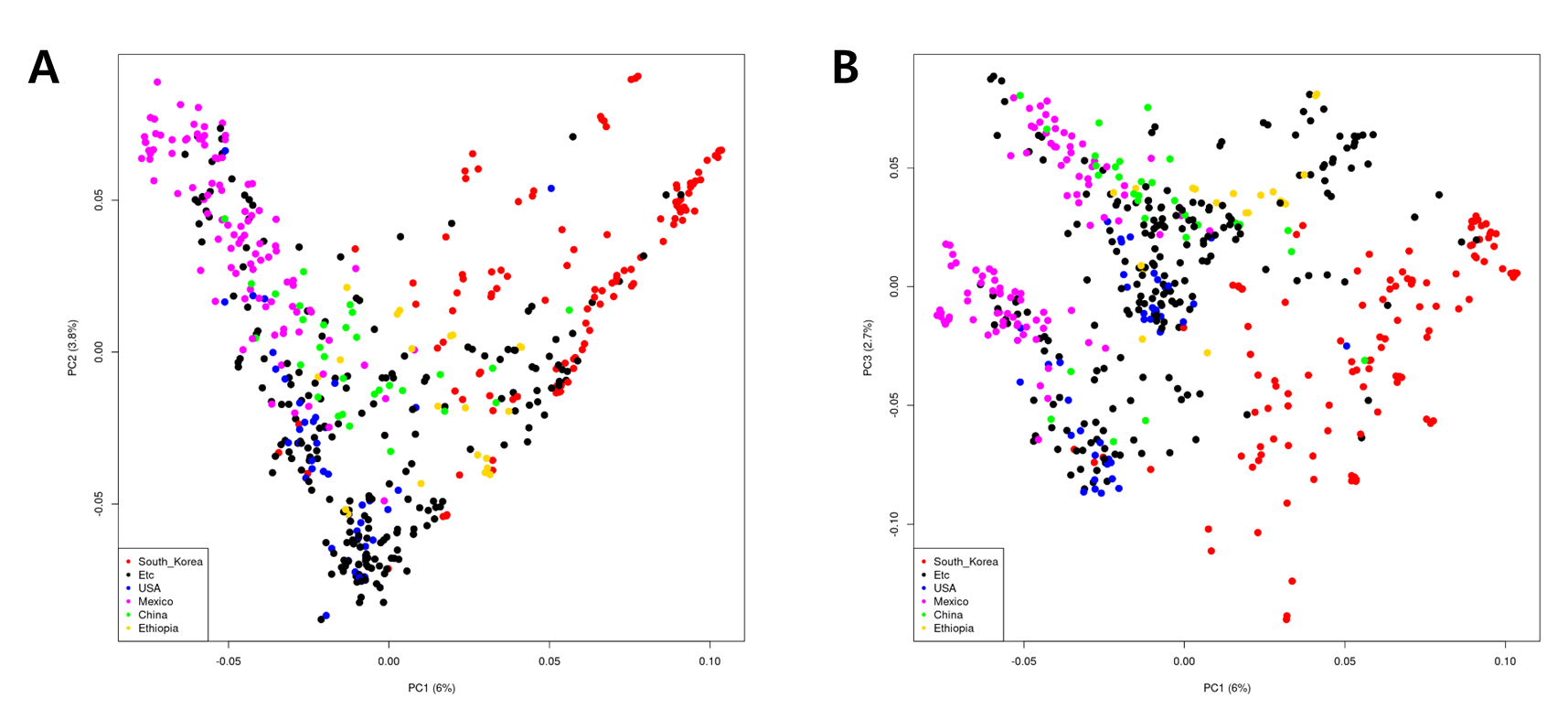

Supplement: S3 Fig — (A) x: PC1, y: PC2, (B) x: PC1, y: PC3. (TIF) [file pone.0322306.s003.tif]

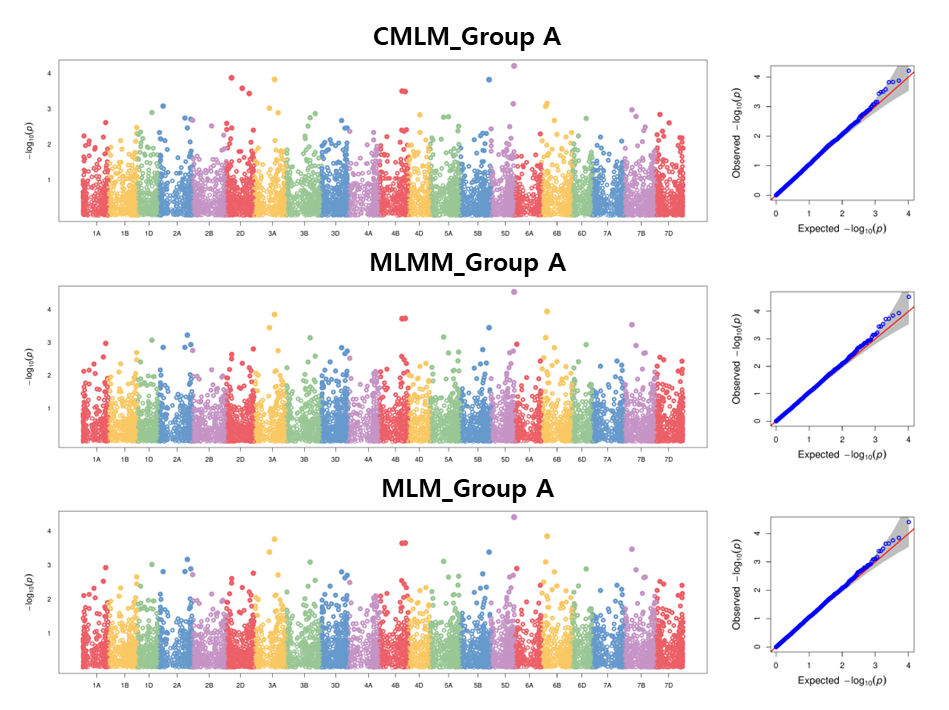

Supplement: S4 Fig — The results correspond to the application of MLM, CMLM, and MLMM to Group A. (TIF) [file pone.0322306.s004.tif]
