# Supplementary material for: Genome-wide association study to identify the genomic loci associated with wheat heading date variation under autumn-sowing conditions
Source: PLoS One. 2025 Apr 30;20(4):e0322306. doi: 10.1371/journal.pone.0322306 (PMC12043121; doi:10.1371/journal.pone.0322306)
Supplement: S1 Table — (DOCX) [file pone.0322306.s005.docx]

**S1 Table. Genetic accessions used in this study according to the country of origin.**

| **No.** | **Country** | **No. of accessions** | **No.** | **Country** | **No. of accessions** |
| --- | --- | --- | --- | --- | --- |
| 1 | Afghanistan | 13 | 26 | Macedonia | 1 |
| 2 | Argentina | 5 | 27 | Mexico | 101 |
| 3 | Australia | 4 | 28 | Mongolia | 6 |
| 4 | Austria | 1 | 29 | Nepal | 2 |
| 5 | Bosnia and Herzegovina | 3 | 30 | Netherlands | 1 |
| 6 | Brazil | 1 | 31 | Pakistan | 3 |
| 7 | Bulgaria | 8 | 32 | Poland | 1 |
| 8 | Canada | 10 | 33 | Portugal | 8 |
| 9 | China | 31 | 34 | Romania | 2 |
| 10 | Colombia | 3 | 35 | Russia | 12 |
| 11 | Croatia | 4 | 36 | Saudi Arabia | 2 |
| 12 | Egypt | 3 | 37 | Slovenia | 1 |
| 13 | Ethiopia | 22 | 38 | South Africa | 2 |
| 14 | Finland | 1 | 39 | Spain | 6 |
| 15 | France | 4 | 40 | Syria | 2 |
| 16 | Germany | 1 | 41 | Tajikistan | 1 |
| 17 | Greece | 3 | 42 | Tunisia | 1 |
| 18 | Hungary | 6 | 43 | Turkey | 10 |
| 19 | India | 6 | 44 | Ukraine | 6 |
| 20 | Italy | 4 | 45 | United States | 42 |
| 21 | Japan | 9 | 46 | Uzbekistan | 2 |
| 22 | Korea, North | 4 | 47 | Zimbabwe | 1 |
| 23 | Korea, South | 120 |  |  |  |
| 24 | Kyrgyzstan | 1 |  |  |  |
| 25 | Lebanon | 2 |  |  |  |
