# Supplementary material for: Genome-wide association study to identify the genomic loci associated with wheat heading date variation under autumn-sowing conditions
Source: PLoS One. 2025 Apr 30;20(4):e0322306. doi: 10.1371/journal.pone.0322306 (PMC12043121; doi:10.1371/journal.pone.0322306)
Supplement: S2 Table — (DOCX) [file pone.0322306.s006.docx]

**S2 Table. List of the PCR primers used for the analysis of allelic variations in *VRN-1* and *PPD-1*.**

| **Gene** | **Primer** | **Sequence** | **Band size**  **(bp)** | **Allele** | **Feature** | **Reference** |
| --- | --- | --- | --- | --- | --- | --- |
| ***VRN-A1*** | (F) VrnN-FP3 | GTGTGTGTTTGTGGCGAGAG |  |  |  | Whittal et al. (2018) [51] |
|  | (R) VrnN-RP3 | CGAAGGCGTATTGGGGAACA | 926 | *Vrn-A1a* | Spring |  |
|  |  |  | 633 | *Vrn-A1b* | Spring |  |
|  |  |  | 662 | *vrn-A1* | Winter |  |
| ***VRN-B1*** | (F) Intr/B/F | CAAGTGGAACGGTTAGGACA |  |  |  | Fu et al. (2005) [13] |
|  | (R) Intr/B/R4 | CAAATGAAAAGGAATGAGAGCA | 1149 | *vrn-B1* | Winter |  |
| ***VRN-D1*** | (F) Intr1/D/F | GTTGTCTGCCTCATCAAATCC |  |  |  | Fu et al. (2005) [13] |
|  | (R) Intr1/D/R3 | GGTCACTGGTGGTCTGTGC | 1671 | *Vrn-D1* | Spring |  |
|  | (R) Intr1/D/R | AAATGAAAAGGAACGAGAGCG | 997 | *vrn-D1* | Winter |  |
| ***PPD-A1*** | (F) TaPpd-A1prodelF | CGTACTCCCTCCGTTTCTTT |  |  |  | Plotnikov et al. (2024) [52] |
|  | (R) TaPpd-A1prodelR2 | GTTGGGGTCGTTTGGTGGTG | 338 | *Ppd-A1a* | Photoperiod Insensitive |  |
|  | (R) TaPpd-A1prodelR3 | AATTTACGGGGACCAAATACC | 299 | *Ppd-A1b* | Photoperiod Sensitive |  |
| ***PPD-B1*** | (F) TaPpd-B1proinF1 | CAGCTCCTCCGTTTGCTTCC |  |  |  | Beales et al. (2007) [53] |
|  | (R) TaPpd-B1proinR1 | CAGAGGAGTAGTCCGCGTGT | 650 | *Ppd-B1a* | Photoperiod Insensitive |  |
|  |  |  | 312 | *Ppd-B1b* | Photoperiod Sensitive |  |
| ***PPD-D1*** | (F) Ppd-D1_F1 | ACGCCTCCCACTACACTG |  |  |  | Beales et al. (2007) [53] |
|  | (R) Ppd-D1_R2 | CACTGGTGGTAGCTGAGATT | 288 | *Ppd-D1a* | Photoperiod Insensitive |  |
|  | (R) Ppd-D1_R1 | GTTGGTTCAAACAGAGAGC | 414 | *Ppd-D1b* | Photoperiod Sensitive |  |
