# Supplementary material for: Genome-wide association study to identify the genomic loci associated with wheat heading date variation under autumn-sowing conditions
Source: PLoS One. 2025 Apr 30;20(4):e0322306. doi: 10.1371/journal.pone.0322306 (PMC12043121; doi:10.1371/journal.pone.0322306)
Supplement: S3 Table — (DOCX) [file pone.0322306.s007.docx]

**S3 Table. Heritability estimation of days to heading in wheat core collections.**

| **Variation** | **DF** | **SS** | **MS** | **F** | **Heritability** |
| --- | --- | --- | --- | --- | --- |
| **Genotype (G)** | 529 | 104689 | 197.9 | 41.9 | 0.89 |
| **Environment (E)** | 4 | 1405 | 351.2 | 74.4 |  |
| **G x E** | 2116 | 9983 | 4.7 |  |  |

DF: degree of freedom, SS: sum of squares, MS: mean square, F: F-test value, G x E: genotype by environment interaction.
