# Supplementary material for: Genome-wide association study to identify the genomic loci associated with wheat heading date variation under autumn-sowing conditions
Source: PLoS One. 2025 Apr 30;20(4):e0322306. doi: 10.1371/journal.pone.0322306 (PMC12043121; doi:10.1371/journal.pone.0322306)
Supplement: S4 Table — (DOCX) [file pone.0322306.s008.docx]

**S4 Table.** **Grouping of wheat core collections based on the *VRN-1* and *PPD-1* genotypes.**

| **No.** | **Alleles^y^** | | | | | | **No. of accession** | **Days to Heading^z^** |
| --- | --- | --- | --- | --- | --- | --- | --- | --- |
|  | ***VRN***  ***-A1*** | ***VRN***  ***-B1*** | ***VRN***  ***-D1*** | ***PPD***  ***-A1*** | ***PPD***  ***-B1*** | ***PPD***  ***-D1*** |  |  |
| 1 | *v* | *v* | *v* | *b* | *b* | *a* | 60 | 185.6^e^ |
| 2 | *v* | *v* | *V* | *b* | *b* | *a* | 24 | 185.7^e^ |
| 3 | *V* | *v* | *v* | *b* | *b* | *a* | 20 | 185.9^e^ |
| 4 | *v* | *V* | *v* | *b* | *b* | *a* | 28 | 186.3^e^ |
| 5 | *V* | *V* | *V* | *b* | *b* | *a* | 5 | 186.3^e^ |
| 6 | *v* | *V* | *V* | *b* | *b* | *a* | 16 | 186.4^e^ |
| 7 | *V* | *V* | *v* | *b* | *b* | *a* | 10 | 186.9^de^ |
| 8 | *V* | *v* | *V* | *b* | *b* | *a* | 4 | 187.9^cde^ |
| 9 | *v* | *V* | *V* | *b* | *b* | *b* | 3 | 188.3^bcde^ |
| 10 | *v* | *v* | *v* | *a* | *b* | *b* | 4 | 189.4^bcde^ |
| 11 | *v* | *v* | *V* | *b* | *b* | *b* | 12 | 191.2^bcde^ |
| 12 | *V* | *v* | *v* | *b* | *b* | *b* | 17 | 192.6^bcd^ |
| 13 | *V* | *V* | *v* | *b* | *b* | *b* | 21 | 192.7^bcd^ |
| 14 | *v* | *V* | *v* | *b* | *b* | *b* | 32 | 193.3^bc^ |
| 15 | *v* | *v* | *v* | *b* | *b* | *b* | 72 | 194.4^ab^ |
| 16 | *V* | *V* | *V* | *b* | *b* | *b* | 1 | 199.6^a^ |

Gray indicates early-heading allele.

^y^ V: Spring type allele, v: Winter type allele, a: Photoperiod-insensitivity type allele, b: Photoperiod-sensitivity type allele.

^z^ Different letters indicate significant differences at *p < 0.05* level according to Duncan's Multiple Range Test.
