# Supplementary material for: Genome-wide association study to identify the genomic loci associated with wheat heading date variation under autumn-sowing conditions
Source: PLoS One. 2025 Apr 30;20(4):e0322306. doi: 10.1371/journal.pone.0322306 (PMC12043121; doi:10.1371/journal.pone.0322306)
Supplement: S5 Table — (DOCX) [file pone.0322306.s009.docx]

**S5 Table. Distribution and days to heading (DTH) based on the *VRN-1* and *PPD-1* genotypes in the wheat core collections by origin and Korean wheat varieties.**

| **Origin** | | **Korea, South**  (n=120) | | **Mexico**  (n=101) | | **USA**  (n=42) | | **China**  (n=31) | | **Ethiopia**  (n=22) | | **Korean Varieties**  (n=40) | |
| --- | --- | --- | --- | --- | --- | --- | --- | --- | --- | --- | --- | --- | --- |
| **Gene** | **Alelle** | **%** | **DTH** | **%** | **DTH** | **%** | **DTH** | **%** | **DTH** | **%** | **DTH** | **%** | **DTH** |
| ***VRN***  ***-A1*** | ***Vrn-A1*** | 0.8 | 197.4 | 29.7 | 187.0 | 16.7 | 188.5 | 32.3 | 189.7 | 13.6 | 187.8 | 0.0 | - |
|  | ***vrn-A1*** | 96.7 | 183.2 | 66.3 | 186.6 | 83.3 | 193.7^**^ | 64.5 | 185.6 | 86.4 | 186.1 | 100.0 | 178.5 |
|  | unknown | 2.5 | - | 4.0 | - | 0.0 | - | 3.2 | - | 0.0 | - | 0.0 | - |
| ***VRN***  ***-B1*** | ***Vrn-B1*** | 2.5 | 183.5 | 55.4 | 187.0 | 23.8 | 188.9 | 61.3 | 188.5^*^ | 68.2 | 187.1^**^ | 20.0 | 177.2 |
|  | ***vrn-B1*** | 85.0 | 183.3 | 43.6 | 186.3 | 71.4 | 194.4^**^ | 38.7 | 184.3 | 31.8 | 184.5 | 80.0 | 178.9 |
|  | unknown | 12.5 | - | 1.0 | - | 4.8 | - | 0.0 | - | 0.0 | - | 0.0 | - |
| ***VRN***  ***-D1*** | ***Vrn-D1*** | 6.7 | 182.7 | 25.7 | 186.7 | 0.0 | - | 32.3 | 187.3 | 18.2 | 185.2 | 42.5 | 178.4 |
|  | ***vrn-D1*** | 62.5 | 183.9 | 31.7 | 186.7 | 95.2 | 192.9 | 45.2 | 187.9 | 63.6 | 187.2 | 57.5 | 178.6 |
|  | unknown | 30.8 | - | 42.6 | - | 4.8 | - | 22.6 | - | 18.1 | - | 0.0 | - |
| ***PPD***  ***-A1*** | ***Ppd-A1a*** | 0.0 | - | 0.0 | - | 2.4 | 187.0 | 0.0 | - | 0.0 | - | 0.0 | - |
|  | ***Ppd-A1b*** | 100.0 | 183.3 | 99.0 | 186.7 | 97.6 | 193.0 | 100.0 | 186.8 | 100.0 | 186.3 | 100.0 | 178.5 |
|  | unknown | 0.0 | - | 1.0 | - | 0.0 | - | 0.0 | - | 0.0 | - | 0.0 | - |
| ***PPD***  ***-B1*** | ***Ppd-B1a*** | 0.0 | - | 0.0 | - | 0.0 | - | 0.0 | - | 0.0 | - | 22.5 | 180.7^***^ |
|  | ***Ppd-B1b*** | 84.2 | 184.2 | 100.0 | 186.7 | 100.0 | 192.8 | 100.0 | 186.8 | 100.0 | 186.3 | 77.5 | 177.9 |
|  | unknown | 15.8 | - | 0.0 | - | 0.0 | - | 0.0 | - | 0.0 | - | 0.0 | - |
| ***PPD***  ***-D1*** | ***Ppd-D1a*** | 88.3 | 182.7 | 84.2 | 186.5 | 21.4 | 191.3 | 74.2 | 186.3 | 36.4 | 184.7 | 100.0 | 178.5 |
|  | ***Ppd-D1b*** | 10.8 | 188.4^*^ | 13.9 | 188.3 | 73.8 | 193.4 | 22.6 | 189.6 | 50.0 | 187.6^*^ | 0.0 | - |
|  | unknown | 0.9 | - | 2.0 | - | 4.8 | - | 3.2 | - | 13.6 | - | 0.0 | - |

**p < 0.05, **p < 0.01, ***p < 0.001.*
