# Supplementary material for: Genome-wide association study to identify the genomic loci associated with wheat heading date variation under autumn-sowing conditions
Source: PLoS One. 2025 Apr 30;20(4):e0322306. doi: 10.1371/journal.pone.0322306 (PMC12043121; doi:10.1371/journal.pone.0322306)
Supplement: S6 Table — (DOCX) [file pone.0322306.s010.docx]

**S6 Table. Single nucleotide polymorphisms (SNPs; -log_10_(*P*)>5 and MAF>5%) identified through genome-wide association study (GWAS).**

| **Group** | **SNP** | **Chr** | **Pos** | **Model** | **P.value** | **-log_10_(*P*)** | **Alleles** | **MAF(%)** | **Difference of DTH^y^** |
| --- | --- | --- | --- | --- | --- | --- | --- | --- | --- |
| A | AX-94435438 | 7B | 285346921 | FarmCPU | 2.55E-08 | 7.59 | A/G | 36.4 | 0.1 |
| A | AX-94520713 | 3A | 478256902 | BLINK | 3.14E-07 | 6.5 | A/G | 22.1 | 3.2 |
| A | AX-94520713 | 3A | 478256902 | SUPER | 3.14E-07 | 6.5 | A/G | 22.1 | 3.2 |
| A | AX-94649893 | 1D | 176889100 | BLINK | 2.36E-06 | 5.63 | A/T | 32.1 | 0.2 |
| A | AX-94649893 | 1D | 176889100 | SUPER | 2.36E-06 | 5.63 | A/T | 32.1 | 0.2 |
| A | AX-94649893 | 1D | 176889100 | FarmCPU | 9.19E-15 | 14.04 | A/T | 32.1 | 0.2 |
| A | AX-94685526 | 2B | 167798344 | BLINK | 2.12E-06 | 5.67 | C/T | 41.9 | 4.5 |
| A | AX-94685526 | 2B | 167798344 | SUPER | 2.12E-06 | 5.67 | C/T | 41.9 | 4.5 |
| A | AX-94841160 | 3A | 688883958 | BLINK | 5.74E-08 | 7.24 | A/G | 30.4 | 2.2 |
| A | AX-94841160 | 3A | 688883958 | SUPER | 5.74E-08 | 7.24 | A/G | 30.4 | 2.2 |
| A | AX-94841160 | 3A | 688883958 | FarmCPU | 3.10E-07 | 6.51 | A/G | 30.4 | 2.2 |
| A | AX-95176936 | 2A | 734976386 | BLINK | 6.55E-09 | 8.18 | G/T | 40.2 | 2.3 |
| A | AX-95176936 | 2A | 734976386 | SUPER | 6.55E-09 | 8.18 | G/T | 40.2 | 2.3 |
| A | AX-95200992 | 6D | 327207457 | FarmCPU | 9.55E-10 | 9.02 | C/T | 6.8 | 1.2 |
| A | AX-95201612 | 7B | 188415291 | BLINK | 9.99E-07 | 6 | A/G | 18.1 | 2.9 |
| A | AX-95201612 | 7B | 188415291 | SUPER | 9.99E-07 | 6 | A/G | 18.1 | 2.9 |
| A | AX-95222044 | 4B | 598261950 | BLINK | 3.91E-19 | 18.41 | A/G | 28.1 | 4.8 |
| A | AX-95222044 | 4B | 598261950 | SUPER | 3.91E-19 | 18.41 | A/G | 28.1 | 4.8 |
| A | AX-95222044 | 4B | 598261950 | FarmCPU | 4.91E-19 | 18.31 | A/G | 28.1 | 4.8 |
| B | AX-94501110 | 3D | 7553544 | FarmCPU | 1.65E-06 | 5.78 | A/C | 14.3 | 2.6 |
| B | AX-94549735 | 4A | 40879550 | FarmCPU | 1.29E-06 | 5.89 | C/T | 49.4 | 1.7 |
| B | AX-94550996 | 6D | 451158577 | BLINK | 6.03E-09 | 8.22 | C/G | 9.5 | 5.4 |
| B | AX-94550996 | 6D | 451158577 | SUPER | 6.03E-09 | 8.22 | C/G | 9.5 | 5.4 |
| B | AX-94763018 | 2A | 38913049 | BLINK | 3.87E-14 | 13.41 | C/T | 34.6 | 1.4 |
| B | AX-94763018 | 2A | 38913049 | SUPER | 3.87E-14 | 13.41 | C/T | 34.6 | 1.4 |
| B | AX-94763018 | 2A | 38913049 | FarmCPU | 2.70E-13 | 12.57 | C/T | 34.6 | 1.4 |
| B | AX-94970315 | 2B | 26581499 | FarmCPU | 1.70E-10 | 9.77 | A/G | 16.5 | 3.9 |
| B | AX-95169202 | 4A | 24766934 | FarmCPU | 5.59E-06 | 5.25 | A/G | 29.4 | 1.2 |
| B | AX-95169202 | 4A | 24766934 | BLINK | 1.53E-06 | 5.81 | A/G | 29.4 | 1.2 |
| B | AX-95169202 | 4A | 24766934 | SUPER | 1.53E-06 | 5.81 | A/G | 29.4 | 1.2 |

SNPs highlighted in gray indicate the selected SNPs.

^y^ DTH : Days to heading.
