# Supplementary material for: Genome-wide association study to identify the genomic loci associated with wheat heading date variation under autumn-sowing conditions
Source: PLoS One. 2025 Apr 30;20(4):e0322306. doi: 10.1371/journal.pone.0322306 (PMC12043121; doi:10.1371/journal.pone.0322306)
Supplement: S7 Table — (DOCX) [file pone.0322306.s011.docx]

**S7 Table. Distribution and days to heading (DTH) based on selected SNP in the wheat core collections by origin and Korean wheat varieties**

| **Origin** | | **Total**  (n=530) | | **Korea, South**  (n=120) | | **Mexico**  (n=101) | | **USA**  (n=42) | | **China**  (n=31) | | **Ethiopia**  (n=22) | | **Korean Varieties**  (n=37) | |
| --- | --- | --- | --- | --- | --- | --- | --- | --- | --- | --- | --- | --- | --- | --- | --- |
| **SNP** | **Allele** | **%** | **DTH** | **%** | **DTH** | **%** | **DTH** | **%** | **DTH** | **%** | **DTH** | **%** | **DTH** | **%** | **DTH** |
| **AX-95222044** | **G** | 28.5 | 185.2 | 59.2 | 182.3 | 5.0 | 187.6 | 26.2 | 189.9 | 25.8 | 185.1 | 27.3 | 184.6 | 67.6 | 178.5 |
|  | **A** | 69.8 | 190.0^***z^ | 36.7 | 185.2^**^ | 95.0 | 186.6 | 73.8 | 193.9^**^ | 74.2 | 187.5 | 72.7 | 186.9 | 32.4 | 177.9 |
|  | **NA** | 1.7 | - | 4.1 | - | 0.0 | - | 0.0 | - | 0.0 | - | 0.0 | - | 0.0 | - |
| **AX-94685526** | **C** | 56.0 | 186.6 | 89.2 | 182.6 | 41.6 | 186.5 | 50.0 | 192.0 | 58.1 | 184.9 | 54.5 | 186.8 | 86.5 | 178.6^**^ |
|  | **T** | 42.8 | 191.1^***^ | 10.0 | 189.0^**^ | 57.4 | 186.8 | 50.0 | 193.6 | 38.7 | 189.7^**^ | 40.9 | 184.9 | 13.5 | 176.6 |
|  | **NA** | 1.2 | - | 0.8 | - | 1.0 | - | 0.0 | - | 3.2 | - | 4.5 | - | 0.0 | - |
| **AX-94550996** | **C** | 9.1 | 187.6 | 12.5 | 177.5 | 5.0 | 185.6 | 4.8 | 201.6 | 3.2 | 173.4 | 0.0 | - | 40.5 | 177.3 |
|  | **G** | 87.2 | 188.9 | 78.3 | 184.6^***^ | 92.1 | 186.7 | 92.9 | 192.4 | 96.8 | 187.3 | 95.5 | 186.4 | 59.5 | 179.0^*^ |
|  | **NA** | 3.8 | - | 9.2 | - | 3.0 | - | 2.4 | - | 0.0 | - | 4.5 | - | 0.0 | - |
| **AX-94970315** | **A** | 74.7 | 187.6 | 73.3 | 181.9 | 92.1 | 186.7 | 61.9 | 192.2 | 100.0 | 186.8 | 68.2 | 185.1 | 91.9 | 178.0 |
|  | **G** | 21.9 | 191.6^***^ | 25.0 | 187.1^***^ | 6.9 | 187.0 | 38.1 | 193.9 | 0.0 | - | 22.7 | 188.5^*^ | 8.1 | 182.3^*^ |
|  | **NA** | 3.4 | - | 1.7 | - | 1.0 | - | 0.0 | - | 0.0 | - | 9.1 | - | 0.0 | - |

^y^ NA (not available).

^z^ **p < 0.05, **p < 0.01, ***p < 0.001.*
