# Supplementary material for: Genome-wide association study to identify the genomic loci associated with wheat heading date variation under autumn-sowing conditions
Source: PLoS One. 2025 Apr 30;20(4):e0322306. doi: 10.1371/journal.pone.0322306 (PMC12043121; doi:10.1371/journal.pone.0322306)
Supplement: S8 Table — (DOCX) [file pone.0322306.s012.docx]

**S8 Table. Grouping of Korean wheat varieties based on the selected SNP genotypes.**

| **No.** | **AX-**  **9522**  **2044** | **AX-**  **9468**  **5526** | **AX-**  **9455**  **0996** | **AX-**  **9497**  **0315** | **Korean Varieties** | **Wheat core collections** |
| --- | --- | --- | --- | --- | --- | --- |
|  |  |  |  |  | **Days to Heading^y^**  **/ No. of accession** | **Days to Heading**  **/ No. of accession** |
| 1 | A | C | G | A | 176^c^ / 3 | 186 / 66 |
| 2 | G | T | C | A | 176^bc^ / 4 | - |
| 3 | G | C | C | A | 177 ^bc^ / 4 | 179 / 5 |
| 4 | A | T | G | A | 177 ^bc^ / 1 | 187 / 72 |
| 5 | A | C | C | A | 178 ^bc^ / 7 | 180 / 13 |
| 6 | G | C | G | A | 179 ^bc^ / 15 | 183 / 59 |
| 7 | G | C | G | G | 181 ^ab^ / 2 | 184 / 5 |
| 8 | A | C | G | G | 185 ^a^ / 1 | 186 / 22 |

Gray indicates alleles associated with early heading.

**^y^** Different letters indicate significant differences at *p < 0.05* level according to Duncan's Multiple Range Test.
