# Supplementary material for: Genome-wide association study to identify the genomic loci associated with wheat heading date variation under autumn-sowing conditions
Source: PLoS One. 2025 Apr 30;20(4):e0322306. doi: 10.1371/journal.pone.0322306 (PMC12043121; doi:10.1371/journal.pone.0322306)
Supplement: Attachment — Submitted filename: PlosOne-Review.docx [file pone.0322306.s013.docx]

Genome-wide association study to identify the genomic loci associated with wheat heading date variation under autumn-sowing conditions

The study evaluated the heading date variation of 530 wheat accessions of various origins under 92 autumn-sowing conditions in Korea for five years.

In general, the manuscript would be of limited interest, as the findings are supportive of well known information. The study identified a percentage of alleles at Vrn and Ppd loci that were listed as ‘unknown’ and with no record of the associated DTH. These could represent rare alleles and should be investigated further to deliver quality science, otherwise the content of paper is only of regional interest.
