# Supplementary material for: Genome-wide association study to identify the genomic loci associated with wheat heading date variation under autumn-sowing conditions
Source: PLoS One. 2025 Apr 30;20(4):e0322306. doi: 10.1371/journal.pone.0322306 (PMC12043121; doi:10.1371/journal.pone.0322306)
Supplement: Attachment — Submitted filename: flowwering time_Manuscript (1).docx [file pone.0322306.s014.docx]

Genome-wide association study to identify the genomic loci associated with wheat heading date variation under autumn-sowing conditions

Short title: GWAS on wheat heading date under autumn-sowing conditions

Yurim Kim^1^, Myoung-Goo Choi^1^, Myoung Hui Lee^1^, Chuloh Cho^1^, Jun Yong Choi^2^, Suk-Jin Kim^1^**,** Chon-Sik Kang^1^, Chul Soo Park^3^, Ki-Chang Jang^1^, Youngjun Mo^3*^, Changhyun Choi^1*^

^1^National Institute of Crop Science, Wanju, 55365, Republic of Korea

^2^ Department of Central Area Crop Science, National Institute of Crop Science, Suwon, 16429, Republic of Korea
^3^Department of Crop Science and Biotechnology, Jeonbuk National University, Jeonju, 54896, Republic of Korea

^*^Corresponding author. E-mail: [chchhy@korea.kr](mailto:chchhy@korea.kr) (CC), [yjmo@jbnu.ac.kr](mailto:yjmo@jbnu.ac.kr) (YM)

**Abstract**

This study aimed to identify genetic loci associated with days to heading (DTH) in wheat under autumn sowing conditions in Korea, where early heading is critical due to the overlap between the wheat harvest and the rainy season. We evaluated 530 wheat core collections over five years, focusing on known heading date genes *VRN-1* and *PPD-1*, and conducted a genome-wide association study (GWAS) to discover new genetic loci related to DTH. The results showed that Korean accessions had the earliest DTH, with modern Korean varieties heading even earlier, reflecting a strong breeding focus on early heading. Among the existing heading date genes, only *PPD-D1* was significantly associated with DTH in the wheat core collection. However, since all Korean varieties carried the same *PPD-D1* allele, the variation in DTH could not be fully explained byPPD. GWAS identified nine SNPs associated with DTH in Group A (the entire collection) and six in Group B (how many? accessions with genotypes identical to Korean varieties). Four key SNPs—two from each group—were selected based on their effect sizes on DTH. In both groups, pyramiding the alleles for early heading was effective in advancing DTH. The findings suggest that the selected SNPs, particularly those reflecting the genotypes of Korean varieties, are effective in explaining DTH variation of Korean varieties and could enhance wheat breeding efficiency in Korea. Further research is needed to validate these loci and identify the underlying genes, which would provide valuable markers for developing early heading wheat varieties suited to Korean autumn-sowing conditions.

**Introduction**

The heading date is a critical factor in the formation and development of wheat seeds, significantly affecting grain yield and quality. Among numerous genetic factors controlling flowering, genes involved in vernalization (*VRN*), photoperiod (*PPD*), and earliness per se (*Eps*) have been studied extensively in wheat [1-2]. Regarding wheat heading date variations, it is estimated that *VRN*, *PPD*, and *Eps* genes contribute approximately 70–75%, 20–25%, and 5%, respectively [3-4].

To date, four *VRN* genes (*VRN-1*, *VRN-2*, *VRN-3*, and *VRN-4*) and two *PPD* genes (*PPD-1* and *PPD-2*) have been identified [5-9]. The *VRN-A1, VRN-B1*, and *VRN-D1* genes are located on chromosomes 5A, 5B, and 5D, respectively. The *VRN-A2* gene was mapped to chromosome 5A, while the *VRN-A3, VRN-B3*, and *VRN-D3* genes are located on chromosomes 7A, 7B, and 7D, respectively. Among the *VRN-4*, *VRN-D4* is located on chromosome 5D [10-16]. *VRN-1* shows greater variability in response to vernalization than other genes, and *VRN-A1* has a higher responsiveness than *VRN-B1* or *VRN-D1* [1,17]. Spring wheat and facultative wheat do not respond to vernalization; however, winter wheat requires vernalization. Spring wheat carries one or more dominant alleles in *VRN-1*, *VRN-3*, and *VRN-4*, whereas winter wheat carries a dominant allele in the *VRN-2* gene and has recessive alleles (*vrn-1*, *vrn-3*, and *vrn-4*) in other loci [1,7,9,18]. The *PPD-A1, PPD-B1*, and *PPD-D1* genes are located on chromosomes 2A, 2B, and 2D, respectively. Among the *PPD-2* genes, *PPD-B2* is located on chromosome 7BS. Photoperiod response is the strongest for *PPD-D1*, followed by *PPD-B1* and *PPD-A1*. The photoperiod-insensitive type with dominant alleles (a-type) heads as soon as the temperature rises in spring, whereas the photoperiod-sensitive type with recessive alleles (b-type) heads only when the day length is sufficiently long [8,9,19-24]. The heading date of spring wheat is significantly influenced by the allelic form of *VRN-1*, whereas that of winter wheat is greatly influenced by the allelic forms of *PPD-D1* and *PPD-B1* [1,25-27]. The *Eps* gene affects heading time in the absence of the effects of vernalization and photoperiod genes. Recently, studies on *EARLY FLOWERING 3 (ELF3)*, a component of the evening complex (EC) in the circadian clock, have shown that *ELF3* loss-of-function results in early heading under both long day and short day conditions [28-32].

In addition to the *VRN* and *PPD* genes, active research has been conducted to explore quantitative trait loci (QTLs) for wheat heading date [9,33-35]. Most QTL studies using bi-parental mapping populations such as F_2_ or recombinant inbred lines are dependent on relatively narrow genetic diversity segregating from the two parental lines. In contrast, genome-wide association studies (GWAS) with diversity panels constitute a useful method for constructing a high-resolution genetic map in a relatively short time using resources with diverse genetic compositions [36-38]. To date, many single nucleotide polymorphism (SNP) markers associated with the heading date variation have been reported on most of the wheat chromosomes [13-14,34-35,39-46].

In Korea experiencing a summer monsoon climate, the wheat harvesting season may overlap with the rainy season [11,47]. Frequent rainfall before the wheat harvest can lead to preharvest sprouting and lodging, causing delays in harvesting operations and potential quality degradation [48]. Therefore, to ensure stable yield and quality of wheat, advancing the heading date would be necessary to expedite maturation without reducing the duration of the maturation phase. To enhance the selection efficiency of wheat breeding programs for the development of early maturing varieties, analysis of the genetic factors controlling heading date variation under the conditions of autumn sowing, prevalent in these regions, would be necessary. In this study, we evaluated the heading date variation of 530 wheat accessions of various origins under autumn-sowing conditions in Korea for five years. Through determining the allele types of *VRN-1* and *PPD-1* and conducting GWAS analysis, we identified new genetic loci associated with the heading date, providing useful information for breeding early heading wheat varieties suitable for the Korean climate under autumn-sowing conditions.

**Materials and Methods**

**Plant materials**

This study used 530 wheat genetic resources and 40 Korean varieties as experimental materials. The 530 accessions belong to a wheat core collection previously built for genomic analysis [49]. Among these accessions, 120 were collected from Korea, and 362 were collected from 47 countries including Mexico (101), the USA (42), China (31), Ethiopia (22), Afghanistan (13), Russia (12), Canada (10), and Turkey (10), while the origin of 48 accessions was unknown (S1 Table).

**Heading date investigation**

The plant materials were sown on October 25th each year from 2018 to 2022 at the National Institute of Crop Science (Jeollabuk-do, Republic of Korea) and cultivated according to the Rural Development Administration (RDA) standard cultivation method [50]. They were grown according to the ear-to-row method with each accession planted in three 1.5 m rows spaced by 0.4 m. The fertilization was based on N:P_2_O_5_:K_2_O = 9.1:7.4:3.9 kg/10a. P_2_O_5_ and K_2_O were applied at the time of sowing, while N was divided into two applications, with 40% at sowing and 60% at the regeneration stage. The heading date was determined when approximately 40% of the total number of heads had emerged, and the days to heading (DTH) from sowing were counted.

The average temperature during the test years (2018–2022) was 9.5°C, 0.4°C higher than the long-term average (1991–2020). The average temperature from sowing (late October) to the elongation stage (late April) before the heading date was 0.7°C higher than the long-term average (S1 Fig). The average temperatures during the emergence stage (early December) over test years were similar to the long-term average, while those during the regeneration (early February), tillering (mid-March), maximum tillering (early April) and elongation (late April) stages were 0.2°C – 1.8°C higher than the long-term average. The average precipitation from sowing to the elongation stage over test years was 259 mm, 37 mm less than the long-term average.

**Genomic DNA extraction and PCR analysis**

Leaves of plants grown in a greenhouse for three weeks were rapidly frozen in liquid nitrogen and then ground into fine powder using a mortar and pestle, then stored at -70°C. Genomic DNA was extracted from the leaf powder using a plant genomic DNA extraction reagent (Solgent Co., Korea). The extracted genomic DNA was quantified using a Nanodrop 1000 spectrophotometer (Thermo Scientific, Wilmington, USA) and stored at -20°C for use. Genotyping analysis of *VRN-A1*, *VRN-B1*, and *VRN-D1* was performed according to the methods of Whittal et al. (2018) [51] and Fu et al. (2005) [13], and *PPD-A1*, *PPD-B1*, and *PPD-D1* were analyzed using the methods of Nishida et al. (2013) [52] and Beales et al. (2007) [53] (S2 Table).

**Genome-wide Association Studies (GWAS)**

For GWAS, genotype data of the 530 wheat accessions from the 35K SNP iSelect chip array (Thermo Fisher Scientific Inc) was utilized [54]. From the initial 35,143 SNPs, 3,218 that are not located on the chromosomes of the IWGSC v1.0 reference (CerealsDB) were excluded. To utilize only high-quality SNPs for GWAS, the filtering process was conducted by the method of Marees et al. (2018) [55]. To minimize redundancy, SNPs were excluded when the calculated R^2^ value between markers within a 50 kb region exceeded 0.2 through LD pruning [56]. Also, accessions with a heterozygosity ratio significantly higher than the average, exceeding +3 standard deviations, were excluded from the analysis. Ultimately, 10,152 SNPs and 518 accessions were used for principal component analysis (PCA) and GWAS.

The initial GWAS was conducted using 518 accessions (Group A). Subsequently, a second GWAS was performed using only 231 accessions (Group B) that possess the *vrn-A1*, *Ppd-A1b*, and *Ppd-D1a* alleles (the same allelic composition as Korean wheat varieties). GWAS was conducted using Genome Association and Prediction Integrated Tool (GAPIT) version 3 [57], applying six methods: Mixed Linear Model (MLM), Compression MLM (CMLM), Bayesian-information and Linkage-disequilibrium Iteratively Nested Keyway (BLINK), Multi-Locus Mixed-Model (MLMM), Fixed and random model Circulating Probability Uniform (FarmCPU), and Settlement of MLM Under Progressively Exclusive Relationship (SUPER).

After the GWAS, SNPs with -log_10_(*P*) > 5, MAF (minor allele frequency) > 5%, and an effect size of at least 3.9 days on DTH were selected. Information on candidate genes near the selected SNPs was obtained from Ensembl Plants, and gene expression was verified on the Wheat Expression Browser (RefSeq1.1). Gene functions were also estimated with reference genomes to the Rice Annotation Project and TAIR (The Arabidopsis Information Resource).

**Statistical analysis**

All statistical analyses were performed using R software version 4.3.1 (R Core Team, 2023). For the analysis of heading date phenotypes, Duncan’s multiple range test was conducted using the 'agricolae' package. The 'psych' package was employed to visualize the correlation between data across different years. Broad sense heritability was calculated using the 'variability' package. Analysis of variance (ANOVA) and t-tests were performed using the 'stats' package.

**Results**

**Heading date variation of the core collection and Korean wheat varieties**

In the wheat core collection comprising 530 accessions from 47 countries, the broad-sense heritability of DTH was high at 0.89 (S3 Table). The average DTH during the test years (2018–2022) was 188.6 days, and there was a high correlation (r ≥ 0.95^***^) between the average DTH and the DTH in each test year (S2 Fig). Among the top five countries of origin comprising the wheat core collection, accessions from Korea exhibited the earliest DTH at 183.3 days, followed by those from Ethiopia (186.3 d), China (186.8 d), Mexico (186.7 d) and the USA (192.8 d) (Fig 1). In addition, Korean varieties (n = 40; modern cultivars released in 1976-2016) exhibited an even earlier DTH of 178.5 days, on average, which was 4.8 days earlier than the Korean landrace accessions (n = 120) in the wheat core collection. This distribution of DTH indicated that accelerating heading has been a major goal in Korean wheat breeding programs.

**Fig 1. Distribution of days to heading in the wheat core collection (n = 530) by geographic origin and Korean wheat varieties (n = 40).** Different letters above the boxplots indicate significant differences at *P* < 0.05 level according to Duncan's Multiple Range Test.

**Allelic variations of *VRN-1* and *PPD-1* in the wheat core collection and Korean varieties**

To characterize the allele types of previously known major heading date genes, genotyping experiments were conducted using the molecular markers for *VRN-1* and *PPD-1* (Table 1). In the wheat core collection (n = 530), 410 (77.4%) accessions carried the winter-type *vrn-A1* while 93 (17.5%) accessions carried the spring-type *Vrn-A1*. There was no significant difference in DTH between the accessions carrying *Vrn-A1* and *vrn-A1*. For *VRN-B1*, 333 (62.8%) carried the winter-type *vrn-B1* and 173 (32.6%) carried the spring-type *Vrn-B1*, without a significant difference in DTH between the accessions carrying *Vrn-B1* and *vrn-B1*. For *VRN-D1*, 330 (62.3%) and 84 (15.8%) carried the winter-type *vrn-D1* and the spring-type *Vrn-D1*, respectively, without a significant difference in DTH. For *PPD-A1*, 521 (98.3%) accessions carried the photoperiod-sensitive type *Ppd-A1b* and 4 (0.8%) carried the photoperiod-insensitive type *Ppd-A1a*, with no significant difference in DTH. For *PPD-B1*, no accession was found to carry the photoperiod-insensitive type *Ppd-B1a*, while 506 accessions (95.5%), for which genotype testing was completed, were confirmed to carry the photoperiod-sensitive type *Ppd-B1b*. For *PPD-D1*, 290 (54.7%) carried the photoperiod-insensitive type *Ppd-D1a*, and 203 (38.3%) carried the photoperiod-sensitive type *Ppd-D1b*. The DTH for accessions carrying *Ppd-D1a* was 185.1 days, which was significantly earlier than the 192.8 days for accessions carrying *Ppd-D1b*. Allele types of all six genes (*VRN-A1*, *VRN-B1*, *VRN-D1*, *PPD-A1*, *PPD-B1*, and *PPD-D1*) were determined in 329 out of the 530 accessions, showing 16 different allele combinations (S4 Table). As in the previous results, DTH was significantly earlier for the combinations with *Ppd-D1a*, and no clear trend was observed for *VRN-1* allele combinations. Korean accessions, which exhibited the earliest DTH, had a higher proportion of photoperiod-insensitive *Ppd-D1a* than those of other countries, whereas the proportion of the spring alleles of *VRN-1* was relatively lower (S5 Table). The accessions from USA, which exhibited the latest DTH, had higher proportions of winter-type *vrn-D1* and photoperiod-sensitive *Ppd-D1b* than those of other countries.

**Table 1. Distribution of *VRN-1* and *PPD-1* alleles in the wheat core collection.**

| **Gene** | **Allele** | | **Number of accessions** | | **DTH**  (2018–2022) | |
| --- | --- | --- | --- | --- | --- | --- |
| ***VRN-A1*** | *Vrn-A1* | | 93 (17.5%) | | | 189.9 |
|  | *vrn-A1* | | 410 (77.4%) | | | 188.4 |
|  | unknown | | 27 (5.1%) | | | - |
| ***VRN-B1*** | *Vrn-B1* | | 173 (32.6%) | | | 189.4 |
|  | *vrn-B1* | | 333 (62.8%) | | | 188.3 |
|  | unknown | | 24 (4.5%) | | | - |
| ***VRN-D1*** | *Vrn-D1* | | 84 (15.8%) | | | 187.0 |
|  | *vrn-D1* | | 330 (62.3%) | | | 190.0 |
|  | unknown | | 116 (21.9%) | | | - |
| ***PPD-A1*** | *Ppd-A1a* | | 4 (0.8%) | | | 188.4 |
|  | *Ppd-A1b* | | 521 (98.3%) | | | 189.6 |
|  | unknown | | 5 (0.9%) | | | ^-^ |
| ***PPD-B1*** | *Ppd-B1b* | | 506 (95.5%) | | | 189.0 |
|  | unknown | | 24 (4.5%) | | | - |
| ***PPD-D1*** | *Ppd-D1a* | | 290 (54.7%) | | | 185.1 |
|  | *Ppd-D1b* | | 203 (38.3%) | | | 192.8^***y^ |
|  | unknown | | 37 (7.0%) | | | - |
|  | | Total | | 530 | 188.6 | |

^y ***^ significant at *P* < 0.001.

In 40 Korean varieties, all carried *vrn-A1* for *VRN-A1* (S5 Table). For *VRN-B1*, 32 (80.0%) carried *vrn-B1* and 8 (20.0%) carried *Vrn-B1*, with no significant difference in DTH between the genotypes, similar to the core collection. For *VRN-D1*, 23 (57.5%) carried *vrn-D1* and 17 (42.5%) carried *Vrn-D1*, with no significant difference in DTH between the genotypes, as in the core collection. For *PPD-A1*, all varieties carried *Ppd-A1b*, whereas for *PPD-D1*, all varieties carried *Ppd-D1a*. For *PPD-B1*, 31 (77.5%) carried *Ppd-B1b* and 9 (22.5%) carried *Ppd-B1a*. Contrary to expectations, varieties with photoperiod-sensitive *Ppd-B1b* showed significantly earlier DTH (177.9 days) than those with photoperiod-insensitive *Ppd-B1a* (180.7 days). Korean wheat varieties had higher proportions of spring and photoperiod-insensitive alleles for *VRN-B1, VRN-D1, PPD-B1*, and *PPD-D1* compared to the Korean accessions in the core collection (S5 Table).

**GWAS on days to heading**

PCA was performed on 518 accessions (12 accessions excluded through the genotype filtering) using 10,152 filtered SNPs. The results showed that the first, second, and third principal components (PC1, PC2, and PC3) explained 6.0%, 3.8%, and 2.7% of the total variance, respectively. PCA revealed that Korean accessions clustered distinctly from other accessions, especially distant from Mexican accessions (S3 Fig).

GWAS analyses for DTH were conducted by classifying the core collection into two groups: Group A (n=518) composed of the entire collection, and Group B (n = 231) composed of the accessions carrying the *VRN-1* and *PPD-1* genotypes (*vrn-A1*, *Ppd-A1b*, and *Ppd-D1a*) that are identical to the Korean varieties. As no SNP exceeding the -log_10_(*P*) threshold was detected with the MLM, CMLM, and MLMM models (Fig. 2A), we report the SNPs for DTH identified from the BLINK, SUPER, and FarmCPU models with the threshold of -log_10_(*P*) > 5 (Fig 2, 3, S4 Fig, and S6 Table). In Group A, nine SNPs were detected on chromosomes 1D, 2A, 2 B, 3A, 4 B, 6D, and 7 B. In Group B, six SNPs were detected on chromosomes 2A, 2 B, 3D, 4A, and 6D. Among the SNPs identified in Group B, AX-94763018 (2A: 38,913,049) was located on the short arm of chromosome 2A, in close proximity to *PPD-A1* (*TraesCS2A01G081900*; 2A: 36,936,362–36,938,400). Considering that all Group B accessions carried the *Ppd-A1b* allele, this SNP presented a potential of a new gene for DTH in this region that is different from *PPD-A1*.

**Fig 2. Manhattan plots and QQ plots for different GWAS models.** The results correspond to the application of the BLINK, FarmCPU, and SUPER models to Group A (A) and Group B (B).

**Fig 3. Chromosomal distribution of 15 SNPs significantly associated with days to heading identified by GWAS (-log_10_(*P*) > 5 and MAF > 5%).** Significant SNPs identified in Group A (Blue) and Group B (Red) are indicated, with selected SNPs marked by a highlighter.

To consider the practical impact of DTH loci on phenotypes, SNPs with an effect size (the difference in DTH between the accessions carrying contrasting genotypes) of at least 3.9 days were selected (Table 2 and S6 Table). The effect size of 3.9 days corresponds to Cohen's d of 0.62, which is considered to have a greater than average effect [58-59]. Based on this criterion, AX-95222044 on chromosome 4B and AX-94685526 on chromosome 2B were chosen in Group A. The accessions carrying an allele for early heading at AX-95222044 (G SNP) and AX-94685526 (C SNP) headed 4.8 days and 4.5 days earlier compared to those carrying an allele for late heading at the two SNPs (A SNP and T SNP), respectively (Fig 4). In Group B, AX-94550996 on chromosome 6D and AX-94970315 on chromosome 2B were selected. The accessions carrying an allele for early heading at AX-94550996 (C SNP) and AX-94970315 (A SNP) headed 5.4 days and 3.9 days earlier than those carrying an allele for late heading at the two SNPs (G SNP and G SNP), respectively (Fig 4).

The heading dates of accessions possessing different allele combinations for the selected SNPs were analyzed for each group (Fig. 4E and Fig. 4F). In Group A, accessions carrying the alleles for early heading at both selected SNPs had the earliest DTH of 184.1 days, while accessions carrying the alleles for late heading at both SNPs had the latest DTH of 191.8 days. The DTH of accessions carrying one allele for early heading at either of the two SNPs were both 188.3 days, indicating that pyramiding the alleles for early heading at the two loci could be effective for accelerating DTH. In Group B, accessions carrying the alleles for early heading at both selected SNPs had the earliest DTH of 178.8 days, followed by the accessions carrying one allele for early heading at AX-94970315 (185.0 days) and the accessions carrying the alleles for late heading at both SNPs (187.7 days). However, two accessions carrying one allele for early heading at AX-94550996 (C SNP) exhibited the latest DTH of 194.4 days, indicating the possibility of genetic interaction among the two loci or effects of other heading date genes.

**Table 2. Genetic information of SNPs identified through GWAS.**

| **Accessions** | **SNP** | **Chr** | **Position** | **Model and *p*-value** | | **Allele** |
| --- | --- | --- | --- | --- | --- | --- |
| **Group A** | **AX-95222044** | 4B | 598261950 | BLINK | 3.91E-19 | G/A |
|  |  |  |  | SUPER | 3.91E-19 |  |
|  |  |  |  | FarmCPU | 4.91E-19 |  |
| **Group A** | **AX-94685526** | 2B | 167798344 | BLINK | 2.12E-06 | C/T |
|  |  |  |  | SUPER | 2.12E-06 |  |
| **Group B** | **AX-94550996** | 6D | 451158577 | BLINK | 6.03E-09 | G/C |
|  |  |  |  | SUPER | 6.03E-09 |  |
| **Group B** | **AX-94970315** | 2B | 26581499 | FarmCPU | 1.70E-10 | G/A |

**Fig 4. Distribution of days to heading based on allelic variations of the selected SNPs by analysis group from wheat core collections.** In Group A, SNPs AX-95222044 (A) and AX-94685526 (B) were analyzed, while in Group B, SNPs AX-94550996 (C) and AX-94970315 (D) were analyzed. Additionally, combinations of SNPs within each group were also analyzed (E-F). ^***^ significant at *P* < 0.001. Different letters above the boxplots indicate significant differences at *P* < 0.05 level according to Duncan's Multiple Range Test.

**Distribution and effects of the selected SNPs from GWAS**

Among the top five countries of origin of the wheat core collection, Korean accessions (n = 120) exhibited the highest frequency of SNPs for early heading at AX-95222044 (G SNP), AX-94685526 (C SNP), and AX-94550996 (C SNP) (Fig 5). Compared to the Korean accessions (n=120; landraces) in the core collection, Korean wheat varieties (n=40) showed even higher frequencies of alleles for early heading at AX-95222044 (G SNP), AX-94550996 (C SNP), and AX-94970315 (A SNP).

**Fig 5. Allele distribution of selected SNPs in the wheat core collections (n = 530) by geographic origin and Korean wheat varieties (n = 37).** Black and white indicate alleles for early heading and late heading, respectively.

Effects of the 4 SNPs discovered through GWAS were examined in 40 Korean wheat varieties, as shown in S7 Table. For AX-94550996, 40.5% carried the allele for early heading (C SNP), showing DTH of 177.3 days which was 1.7 days earlier than those carrying the allele for late heading (G SNP). For AX-94970315, 91.9% carried the allele for early heading (A SNP), showing DTH (178.0 days) which is 4.3 days earlier than those carrying the allele for late heading (G SNP). For AX-94685526, 86.5% carried the allele for early heading (C SNP), showing DTH (178.6 days) which is 2.0 days later than those carrying the allele for late heading (T SNP), showing an opposite effect to that in the core collection. For AX-95222044, 67.6% carried the allele for early heading (G SNP), showing DTH (178.5 days) which is 0.6 days later than those carrying the allele for late heading (A SNP), with no significant difference in DTH.

As with the core collection, considering the four SNP genotypes selected through GWAS, the Korean varieties were divided into eight combinations (S8 Table). One combination that was not present in the core collection was identified, and the remaining seven combinations were among the top eight combinations with the earliest heading dates in the core collection. Differences in heading dates occurred between the Korean varieties and core collections for common genotypic combinations, likely because of the small number of accessions in each genotypic combination and their different genetic backgrounds.

**Discussion**

The objective of this study was to explore the genetic loci involved in DTH variation of wheat under autumn sowing conditions in Korea. Using a wheat core collection, we analyzed the genotypes of previously reported major heading date genes and conducted a GWAS. Heritability of the heading date phenotype in the core collection over five years was high, at 0.89, which was consistent with previous findings [60, 61]. This suggested that the use of molecular markers to select early-heading wheat varieties would be efficient. Among the top five countries comprising the wheat core collection investigated herein, Korean accessions composed of landraces had the earliest heading dates, and Korean varieties headed even earlier. This implied that wheat breeding in Korea is directed towards developing early heading cultivars to facilitate the prevalent rice-wheat double cropping system in Korea, and that the alleles for early heading might have accumulated in major heading date genes [62].

In the core collection, the genotypes of *VRN-1* were not significantly associated with DTH variation. It could be due to the fulfillment of vernalization requirements as a result of autumn sowing in the Korean climate, leading to a reduced impact [1,25-26]. Among the *PPD-1* genes, only the genotypes of *PPD-D1* showed a significant effect on DTH in the wheat core collections. This was consistent with the results of Pugsely (1996) [19], which indicated that among the photoperiod-related *PPD-1* genes, *PPD-D1* was the most influential. It was also consistent with the results of Grogan et al. (2016) [27], which indicated that the heading date of winter wheat was more strongly influenced by photoperiod loci than by vernalization loci*.* Wheat exhibits regional variations in *PPD-1* genotype distribution worldwide, as observed in European winter wheat, where the *Ppd-D1a* allele is mainly present in Eastern, Southern European, and Eurasian varieties [63]. In this study, all Korean varieties were found to possess the photoperiod-insensitive *Ppd-D1a* allele associated with earlier heading. This corresponded to previous studies that indicated limited variability in the genetic composition of the heading date-related genes *VRN-1* and *PPD-1* in Korean wheat varieties and accessions, and indicated that previously reported heading-date genes have limited capability of explaining the DTH variation among Korean wheat varieties [62,64-66].

The GWAS conducted on DTH utilized a wheat core collection comprised of genetically diverse accessions suitable for analysis [49,67]. The GWAS of 518 accessions (Group A) and 231 accessions (Group B, possessing the same alleles as the Korean varieties) identified nine and six non-overlapping SNPs which are significantly associated with DTH. Considering the practical impact on DTH, AX-95222044 and AX-94685526 were selected in Group A, and AX-94550996 and AX-94970315 in Group B. The markers AX-95222044 and AX-94970315 have been previously reported to be associated with heading date in wheat. In a study utilizing the wheat core collection, as in this study, GWAS was conducted on various agricultural traits, including heading date [68]. The results revealed that AX-95222044 exhibited a significant association with heading date (-log_10_ (*P*) = 3.09). Additionally, in another study involving a population derived from crosses between 54 Synthetic Hexaploid Wheat (SHW) lines and nested association mapping (NAM) parents, QTL analysis was performed on the heading date phenotype using 35K SNP chip data, identifying QFt.niab-2B.1 with the peak marker AX-94970315 (-log_10_(*P*) = 6.34) [69]. Contrary to our expectations, *PPD-D1* was not identified in GWAS using Group A, despite that *PPD-D1* genotype had significant effects on DTH (Table 1) and that AX-94609980 (2D: 33,953,727) within *PPD-D1* (*TraesCS2D02G079600*; 2D: 33,952,488–33,955,629), was included in the GWAS analysis. This may be due to the skewed genotype distribution of SNP AX-94609980, of which the T SNP was predominant (94.8%) in the GWAS panel, reducing statistical power.

Analysis of the genotype distribution of SNPs selected based on the country of origin in the wheat core collection revealed that among the top five countries comprising the wheat core collection, the Korean accession, which exhibited the earliest heading, showed a higher proportion of genotypes for early heading than accessions from other countries. Moreover, the proportion of genotype with an earlier heading date tended to increase in Korean varieties than in Korean accessions. This suggested that breeding of Korean varieties has advanced the heading date by the accumulation of genetic types with earlier heading of selected SNPs (Fig 5 and S7 Table). Analysis of the selected SNP genotypes in Korean varieties showed that the two SNPs selected through GWAS in Group B, which reflected the genotypes of Korean varieties, were more suitable for explaining the variation in heading dates of the Korean varieties (S7 Table). Therefore, breeding efficiency would be enhanced by utilizing the four selected SNPs along with the existing heading-date gene *PPD-D1* as selection markers under autumn-sowing conditions in Korea. In addition, accessions possessing early-heading alleles for the four SNPs identified through GWAS, and *PPD-D1*, were considered potential candidates for early-heading breeding materials. Five Korean accessions (Korean Genebank’s Identity Numbers (IT nos). 14303, 15864, 116118, 175533, and 237807) met this criterion, with an average DTH of 179 days.

All four SNPs were located in the coding sequence (CDS) of specific genes. AX-95222044, located in the CDS of *TraesCS4B02G307900*, could potentially induce a loss of function owing to the creation of an early stop codon by the A allele. *TraesCS4B02G307900* showed high expression in parts of the spike and during early spike development, booting, and flowering stages, according to the Wheat Expression Browser prediction (data not shown). AX-94685526, located in the CDS of *TraesCS2B02G192300*, induced amino acid changes. However, no specific expression related to heading was observed. AX-94550996, located in the CDS of *TraesCS6D02G358000*, caused amino acid changes depending on the genotype. This gene had high expression in the central axis of leaves and flowers, and its *Arabidopsis* ortholog *AT5G54390* has been reported to influence flowering time [70]. AX-94970315, located in the CDS of *TraesCS2B02G054700*, did not cause amino acid changes based on the genotype. Further research using additional genetic analysis populations would be required to validate the effects of these four heading date associated loci and to identify the causative genes.

**Acknowledgments**

Not applicable.

**References**

1. Kamran A, Iqbal M, Spaner D. Flowering time in wheat (*Triticum aestivum* L.): a key factor for global adaptability. Euphytica. 2014; 197: 1-26.
2. Lee JH. Flowering-time genes and flowering-time pathways in wheat (*Triticum aestivum* L.). Korean J. Breed. Sci. 2019; 51: 65-72.
3. Law CN, Worland AJ. Genetic analysis of some flowering time and adaptive traits in wheat. New Phytol. 1997; 137: 19-28.
4. Stelmakh A. Genetic systems regulating flowering response in wheat. Euphytica. 1998; 100: 359-369.
5. Worland AJ. The influence of the flowering time genes on environmental adaptability in European wheats. Euphytica. 1996; 89: 49-57.
6. Worland AJ, Borner A, Korzun V, Li WM, Petrovic S, Sayers EJ. The influence of photoperiod genes on the adaptability of European winter wheats. Euphytica. 1998; 100: 385-394.
7. Distelfeld A, Li C, Dubcovsky J. Regulation of flowering in temperate cereals. Curr. Opin. Plant Biol. 2009a; 12: 178-184.
8. Khlestkina EK, Giura A, Röder MS, Borner A. A new gene controlling the flowering response to photoperiod in wheat. Euphytica. 2009; 165: 579-585.
9. Shi C, Zhao L, Zhang X, Lv G, Pan Y, Chen F. Gene regulatory network and abundant genetic variation play critical roles in heading stage of polyploidy wheat. BMC Plant Biol. 2019; 19: 6.
10. Dubcovsky J, Lijavetzky D, Appendino L, Tranquilli G, Dvorak JD. Comparative RFLP mapping of *Triticum monococcum* genes controlling vernalization requirement. Theor. Appl. Genet. 1998; 97: 968-975.
11. Iwaki K, Nakagawa K, Kuno H, Kato K. Ecogeographical differentiation in east Asian wheat, revealed from the geographical variation of growth habit and *Vrn* genotype. Euphytica. 2000; 111: 137-143.
12. Yan L, Loukoianov A, Blechl A, Tranquilli G, Ramakrishna W, SanMiguel P, et al. The wheat *VRN2* gene is a flowering repressor down-regulated by vernalization. Science. 2004; 303: 1640-1644.
13. Fu D, Szűcs P, Yan L, Helguera M, Skinner JS, Von Zitzewitz J, et al. Large deletions within the first intron in *VRN-1* are associated with spring growth habit in barley and wheat. Mol. Genet. Genomics. 2005; 273: 54–65.
14. Bonnin I, Rousset M, Madur D, Sourdille P, Dupuits C, Brunel D, et al. FT genome A and D polymorphisms are associated with the variation of earliness components in hexaploid wheat. Theor. Appl. Genet. 2008; 116: 383-394.
15. Distelfeld A, Tranquilli G, Li C, Yan L, Dubcovsky J. Genetic and molecular characterization of the *VRN2* loci in tetraploid wheat. Plant Physiol. 2009b; 149: 245-257.
16. Yoshida T, Nishida H, Zhu J, Nitcher R, Distelfeld A, Akashi Y, et al. *Vrn-D4* is a vernalization gene located on the centromeric region of chromosome 5D in hexaploid wheat. Theor. Appl. Genet. 2010; 120: 543-552.
17. Goncharov NP. Response to vernalization in wheat: its quantitative or qualitative nature. Cereal Res. Comm. 2004; 32: 323-330.
18. Loukoianov A, Yan L, Blechl A, Sanchez A, Dubcovsky J. Regulation of *VRN1* vernalization genes in normal and transgenic polyploid wheat. Plant Physiol. 2005; 138: 2364-2373.
19. Pugsely AT. The photoperiodic sensitivity of some spring wheats with special reference to the variety Thatcher. Aust. J. Agr. Res. 1966; 17: 591-599.
20. Law CN, Sutka J, Worland AJ. A genetic study of day length response in wheat. Heredity. 1978; 41: 575-585.
21. Scarth R, Law CN. The location of the photoperiod gene *Ppd-B1* and an additional genetic factor for ear emergence time on chromosome 2B of wheat. Heredity. 1983; 51: 607-619.
22. Dyck JA, Matus-Cádiz MA, Hucl P, Talbert L, Hunt T, Dubuc JP, et al. Agronomic performance of hard red spring wheat isolines sensitive and insensitive to photoperiod. Crop Sci. 2004; 44: 1976-1981.
23. Bentley AR, Horsnell R, Werner CP, Turner AS, Rose GA, Bedard C, et al. Short, natural, and extended photoperiod response in BC2F4 lines of bread wheat with different *photoperiod-1* (*Ppd-1*) alleles. J. Exp. Bot. 2013; 64: 1783-1793.
24. Zhao YY, Wang X, Wei L, Wang JX, Yin J. Characterization of *Ppd-D1* alleles on the developmental traits and rhythmic expression of photoperiod genes in common wheat. J. Intgr. Agr. 201; 15: 502-11.
25. Iqbal M, Navabi A, Yang RC, Salmon DF, Spaner D. The effect of vernalization genes on earliness and related agronomic traits of spring wheat in northern growing regions. Crop Sci. 2007; 47: 1031-1039.
26. Chen H, Moakhar NP, Iqbal M, Pozniak C, Hucl P, Spaner D. Genetic variation for flowering time and height reducing genes and important traits in western Canadian spring wheat. Euphytica. 2015; 208: 377-390.
27. Grogan SM, Brown-Guedira G, Haley SD, McMaster GS, Reid SD, Smith J, et al. Allelic variation in developmental genes and effects on winter wheat heading date in the U.S. great plains. PLoS One. 2016; 11: e0152852.
28. Zikhali M, Leverington-Waite M, Fish L, Simmonds J, Orford S, Wingen LU, et al. Validation of a 1DL earliness *per se* (*eps*) flowering QTL in bread wheat (*Triticum aestivum*). Mol. Breeding. 2014; 34: 1023-1033.
29. Alvarez MA, Tranquilli G, Lewis S, Kippes N, Dubcovsky J. Genetic and physical mapping of the earliness *per se* locus *Eps-A (m)* 1 in Triticum monococcum identifies *EARLY FLOWERING 3 (ELF3)* as a candidate gene. Funct. Integr. Genomic. 2016; 16: 365-382.
30. Alvarez MA, Li C, Lin H, Joe A, Padilla M, Woods DP, et al. *EARLY FLOWERING 3* interactions with *PHYTOCHROME B* and *PHOTOPERIOD1* are critical for the photoperiodic regulation of wheat heading time. PLoS genet. 2023; 19.5: e1010655.
31. Wittern L, Steed G, Taylor LJ, Ramirez DC, Pingarron-Cardenas G, Gardner K, et al. Wheat *EARLY FLOWERING 3* affects heading date without disrupting circadian oscillations. Plant Physiol. 2023; 191.2: 1383-1403.
32. Mizuno N, Matsunaka H, Yanaka M, Ishikawa G, Kobayashi F, Nakamura K. Natural variations of wheat *EARLY FLOWERING 3* highlight their contributions to local adaptation through fine-tuning of heading time. Theor. Appl. Genet. 2023; 136.6: 139.
33. Zanke C, Ling J, Plieske J, Kollers S, Ebmeyer E, Korzun V, et al. Genetic architecture of main effect QTL for heading date in European winter wheat. Front. Plant Sci. 2014; 5: 217.
34. Hu J, Wang X, Zhang G. Jiang P, Chen W, Hao Y, et al. QTL mapping for yield-related traits in wheat based on four RIL populations. Theor. Appl. Genet. 2020; 133: 917–933.
35. Benaouda S, Dadshani S, Koua P, Léon J, Ballvora A. Identification of QTLs for wheat heading time across multiple-environments. Theor. Appl. Genet. 2022; 135: 2833–2848.
36. Price AL, Patterson NJ, Plenge RM, Weinblatt ME, Shadick NA, Reich D. Principal components analysis corrects for stratification in genome-wide association studies. Nat. Genet. 2006; 38: 904–909.
37. Tshikunde NM, Mashilo J, Shimelis H, Odindo A. Agronomic and physiological traits, and associated quantitative trait loci (QTL) affecting yield response in wheat (*Triticum aestivum* L.): A review. Front. Plant Sci. 2019; 10: 1428.
38. Khan H, Krishnappa G, Kumar S, Mishra CN, Krishna H, Devate NB, et al. Genome-wide association study for grain yield and component traits in bread wheat (*Triticum aestivum* L.). Frontiers in Genetics. 2022; 13: 982589.
39. Chu CG, Xu SS, Friesen TL. Faris JD. Whole genome mapping in a wheat doubled haploid population using SSRs and TRAPs and the identification of QTL for agronomic traits. Mol. Breeding. 2008; 22: 251–266.
40. Zhang K, Zhang Y, Chen G, Yian J. Genetic analysis of grain yield and leaf chlorophyll content in common wheat. Cereal Res. Comm. 2009; 37: 499–511.
41. Bogard M, Jourdan M, Allard V, Martre P, Perretant MR, Ravel C, et al. Anthesis date mainly explained correlations between post-anthesis leaf senescence, grain yield, and grain protein concentration in a winter wheat population segregating for flowering time QTLs. J. Exp. Bot. 2011; 62: 3621–3636.
42. Le Gouis J, Bordes J, Ravel C, Heumez E, Faure S, Praud S, et al. Genome-wide association analysis to identify chromosomal regions determining components of earliness in wheat. Theor. Appl. Genet. 2012; 124: 597-611.
43. Yu K, Liu D, Wu W, Yang W, Sun J, Li X, et al. Development of an integrated linkage map of einkorn wheat and its application for QTL mapping and genome sequence anchoring. Theor. Appl. Genet. 2017; 130: 53-70.
44. Li F, Wen W, He Z, Liu J, Jin H, Cao S, et al. Genome-wide linkage mapping of yield-related traits in three Chinese bread wheat populations using high-density SNP markers. Theor. Appl. Genet. 2018; 131: 1903-1924.
45. Chen Z, Cheng X, Chai L, Wang Z, Du D, Wang Z, et al. Pleiotropic QTL influencing spikelet number and heading date in common wheat (*Triticum aestivum* L.). Theor. Appl. Genet. 2020; 133: 1825-1838.
46. Chen Z, Ke W, He F, Chai L, Cheng X, Xu H, et al. A single nucleotide deletion in the third exon of *FT-D1* increases the spikelet number and delays heading date in wheat (*Triticum aestivum* L.). Plant Biotechnol. J. 2022; 20: 920-933.
47. Yihui D, Chan JC. The East Asian summer monsoon: an overview. Meteorol. Atmos. Phys. 2005; 89.1: 117-142.
48. Simsek S, Ohm JB, Lu H, Rugg M, Berzonsky W, Alamri MS, et al. Effect of pre‐harvest sprouting on physicochemical changes of proteins in wheat. J. Sci. Food Agr. 2014; 94.2: 205-212.
49. Min KD, Kang YN, Kim CS, Choi CH, Kim JY. Genetic diversity and population structure of Korean common wheat (*Triticum aestivum*). Korean J. Breed. Sci. 2021; 53: 277-88.
50. Rural Development Administration (RDA). Standard of research and analysis for agricultural technology. RDA; 2012.
51. Whittal A, Kaviani M, Graf R, Humphreys G, Navabi A. Allelic variation of vernalization and photoperiod response genes in a diverse set of north American high latitude winter wheat genotypes. Plos one. 2018; 13: e0209543.
52. Nishida H, Yoshida T, Kawakami K, Fujita M, Long B, Akashi Y, et al. Structural variation in the 5′ upstream region of photoperiod-insensitive alleles *Ppd-A1a* and *Ppd-B1a* identified in hexaploid wheat (*Triticum aestivum* L.), and their effect on heading time. Mol. Breeding. 2013; 31: 27-37.
53. Beales J, Turner A, Griffiths S, Snape JW, Laurie DA. A pseudo-response regulator is misexpressed in the photoperiod insensitive *Ppd-D1a* mutant of wheat (*Triticum aestivum* L.). Theor. Appl. Genet. 2007; 115: 721–33.
54. Allen AM, Winfield MO, Burridge AJ, Downie RC, Benbow HR, Barker GL, et al. Characterization of a Wheat Breeders’ Array suitable for high‐throughput SNP genotyping of global accessions of hexaploid bread wheat (*Triticum aestivum*). Plant Biotechnol. J. 2017; 15.3: 390-401.
55. Marees AT, de Kluiver H, Stringer S, Vorspan F, Curis E, Marie-Claire C, et al. A tutorial on conducting genome-wide association studies: Quality control and statistical analysis. Int. J. Methods Psychiatr. Res. 2018; 27: e1608.
56. Allen AM, Winfield MO, Burridge AJ, Downie RC, Benbow HR, Barker GL, et al. Characterization of a wheat breeders' array suitable for high-throughput SNP genotyping of global accessions of hexaploid bread wheat (*Triticum aestivum*). Plant Biotechnol. J. 2017; 15: 390-401.
57. Wang J, Zhang Z. GAPIT Version 3: boosting power and accuracy for genomic association and prediction. Genomics Proteomics Bioinformatics. 2021; 19: 629-640.
58. Cohen J. Statistical power for the behavioural sciences. Hilsdale. NY:Lawrence Erlbaum. 1988; 58.1: 7-19.
59. Leppink J, O’Sullivan P, Winston K. Leppink, J., O’Sullivan, P., & Winston, K. (2016). Effect size–large, medium, and small. Perspect. Med. Educ. 2016; 5:347-349.
60. Snape JW, Butterworth K, Whitechurch E, Worland AJ. Waiting for fine times: genetics of flowering time in wheat. Euphytica. 2001; 119: 185-190.
61. Slafer GA. Genetic basis of yield as viewed from a crop physiologist's perspective. Ann. Appl. Biol. 2003; 142.2: 117-128.
62. Cho EJ, Kang CS, Jung JJ, Yoon YM, Park CS. Allelic variation of *Rht-1, Vrn-1 and Ppd-1* in Korean wheats and its effect on agronomic traits. Plant Breeding Biotechnology. 2015a; 3: 129-138.
63. Langer SM, Longin CFH, Würschum T. Flowering time control in European winter wheat. Front. Plant Sci. 2014; 5: 112023.
64. Cho EJ, Kang CS, Yoon YM, Park CS. The relationship between allelic variations of *Vrn-1* and *Ppd-1* and agronomic traits in Korean wheat cultivars. Indian J. Genet. P.L. B.R. 2015b; 75: 294-300.
65. Lee HS, Jung JU, Kang CS. Heo HY, Park CS. Mapping of QTL for yield and its related traits in a doubled haploid population of Korean wheat. Plant Biotechnol. Rep. 2014; 8: 443–454.
66. Cho SW, Kang TG, Kang SW, Kang CS, Park CS. Assessment of DNA markers related to days to heading date, tiller number, and yield in Korean wheat populations. Korean J. Breed. Sci. 2018; 50: 211-223.
67. Lee MH, Park J, Kime KH, Kim KM, Kang CS, Lee GE, et al. Genome-wide association study of arabinoxylan content from a 562 hexaploid wheat collection. Plants. 2023; 12.1: 184.
68. Kang Y, Choi C, Kim JY, Min KD, Kim C. Optimizing genomic selection of agricultural traits using wheat core collection. Front. Plant Sci. 2023; 14: 1112297.
69. Wright TI, Horsenll R, Love B, Burridge AJ, Gardner KA, Jackson, et al. A new winter wheat genetic resource harbours untapped diversity from synthetic hexaploidy wheat. Theor. Appl. Genet. 2024; 137.3: 1-19.
70. Kim BH, von Arnim AG. *FIERY1* regulates light-mediated repression of cell elongation and flowering time via its 3'(2'),5'-bisphosphate nucleotidase activity. The Plant Journal. 2009; 58.2: 208-19.

**Supporting information**

**S1 Fig. Mean temperature and precipitation over the test years (2018–2022).** and indicate the mean temperature and precipitation for the test years, and and indicate the mean temperature and precipitation for a long-term average (1991–2020). I, II, III, IV, V, VI, and VII represent the emergence, regeneration, tillering, elongation, heading, milky, and maturation stages, respectively.

**S2 Fig.** **Correlation between test year days to heading (2018–2022) and their average.** (A) Wheat core collections (n = 530). (B) Korean wheat varieties (n = 40). ^***^ significant at *P* < 0.001.

**S3 Fig. PCA results and the distribution of** **accessions of wheat core collections.** (A) x: PC1, y: PC2, (B) x: PC1, y: PC3.

**S4 Fig. Manhattan plots and QQ plots for different GWAS models.** The results correspond to the application of MLM, CMLM, and MLMM to Group A.

**S1 Table. Genetic** **accessions used in this study according to the country of origin.**

**S2 Table. PCR primers for the analysis of allelic variations in *VRN-1* and *PPD-1*.**

**S3 Table. Heritability estimation of days to heading in wheat core collections.**

**S4 Table.** **Grouping of wheat core collections based on *VRN-1* and *PPD-1* genotypes.**

**S5 Table. Distribution and days to heading (DTH) based on *VRN-1* and *PPD-1* in the wheat core collections by origin and Korean wheat varieties.**

**S6 Table. SNPs (-log_10_(*P*) > 5 and MAF > 5%) identified through GWAS.**

**S7 Table. Distribution and days to heading (DTH) based on selected SNP in the wheat core collections by origin and Korean wheat varieties.**

**S8 Table. Grouping of Korean wheat varieties based on the selected SNP genotypes.**
