# Supplementary material for: Genome-wide association study to identify the genomic loci associated with wheat heading date variation under autumn-sowing conditions
Source: PLoS One. 2025 Apr 30;20(4):e0322306. doi: 10.1371/journal.pone.0322306 (PMC12043121; doi:10.1371/journal.pone.0322306)
Supplement: Attachment — Submitted filename: Response to Reviewers.docx [file pone.0322306.s016.docx]

**Response to Reviewer’s comments**

We sincerely appreciate the reviewer’s valuable comments, which have greatly contributed to improving the quality of our manuscript. Below, we provide our response to the reviewer’s comments.

**Major revisions**

1. In general, the manuscript would be of limited interest, as the findings are supportive of well known information. The study identified a percentage of alleles at Vrn and Ppd loci that were listed as ‘unknown’ and with no record of the associated DTH. These could represent rare alleles and should be investigated further to deliver quality science, otherwise the content of paper is only of regional interest.

- As the reviewer pointed out, our study deals with well-known loci, which may limit its novelty. However, our study employed a differentiated strategy to identify new genetic markers influencing heading date beyond the well-known loci using GWAS. Specifically, we selected resources from a wheat core set that had the same VRN1 and PPD1 genotypes as those of Korean wheat varieties, which are known globally for their early heading characteristics. Using this approach, we were able to discover new SNP markers associated with heading date variation. Additionally, we revised the Abstract and Discussion sections of the manuscript to better highlight this differentiated methodology.
- I believe that this paper not only proposes an innovative experimental approach in GWAS analysis but also provides valuable information for the development of more effective heading date markers applicable to countries such as Japan and China.
- Despite repeated PCR experiments performed multiple times to confirm the *Vrn* and *Ppd* genotypes, resources with unresolved genotypes are still classified as 'unknown.' I believe this issue cannot be resolved using the same approach. To address this problem, it will be necessary to sequence the full nucleotide sequences of the *Vrn* and *Ppd* loci and design various primer combinations based on this information. This could present a new and independent challenge.

2. The paper is well written. The introduction sets out the reasons for the study, but the methods and data do not adequately answer the questions being posed. A high percentage of alleles at known genetic loci for heading date in wheat are described as unknown. These should be further investigated, as they could represent novel alleles or genes, and would add quality to the manuscript. I suggest to do this and resubmit.

- I agree with the reviewer's opinion. Therefore, we conducted a statistical analysis of the number of days to heading by allele for the newly discovered genetic marker loci, as well as for each of the already known heading genes (*Vrn* and *Ppd*), as presented in Table 1 and Table 2, respectively, in line with the reviewer’s suggestion. Additionally, we incorporated explanations into the Results and Discussion sections, highlighted in blue text.

**Minor revisions**

1. 2 page 32 line : Pl present data

Response: Corrected

2. 2 page 36 line : Which loci-describe here

Response: Specifically provided information about the loci in the abstract.

3. 3 page 56 line : Describe this

Response: Corrected

4. 5 page 99 line : 40 are part of 530?

Response: Indicated that the 530 wheat core collection resources and the 40 Korean varieties are separate.

5. 5 page 101 line : This does not equates to 362

Response: The numbers provided in the manuscript represent the major contributors to the 362 accessions collected from 47 countries. Smaller contributions from other countries were not explicitly listed to maintain conciseness, but they are included in the total count of 362 accessions.

6. 5 page 110 line : Not self explanatory

Response: Corrected

7. 6 page 113 line : What this means, regeneration stage?

Response: Added explanations for the terms.

8. 7 page 138 line : Have authors used *VRN-A1*, *VRN-B1*, and *VRN-D1* data for GWAS?

Response: The *VRN-1* data was indirectly utilized in GWAS, as the second GWAS (Group B) was performed using accessions that possess the *vrn-A1* allele, reflecting the allelic composition of Korean wheat varieties.

9. 7 page 145 line : What were the values for heterozygosity? + and - 3 SD?

Response: Provided the values.

10. 11 page table : Average?, Author should have conducted MET analysis

Response: Reanalyzed the data to present stability index by genotype and revised the relevant content in the manuscript accordingly.

11.  page 237 line : What this means, in relation to population structure?

Response: The percentages of the principal components (PC1, PC2, and PC3) indicate the proportion of total genetic variation explained by each component. PC1, explaining 6.0% of the variance, captures the primary axis of genetic variation, while PC2 and PC3, explaining 3.8% and 2.7% respectively capture additional axes of variation. These values reflect the genetic structure within the population.

12. 14 page 273 line : ?

Response: Corrected

13. 15 page table : Provide R2 values-phenotypic or genetic variance accounted

Response: Added proportion of variance explained (PVE).

14. 30 page 402 line : data not shown

Response: Corrected

In addition, some revisions were made to improve the clarity of the manuscript.
