# Supplementary material for: Genome-wide association study to identify the genomic loci associated with wheat heading date variation under autumn-sowing conditions
Source: PLoS One. 2025 Apr 30;20(4):e0322306. doi: 10.1371/journal.pone.0322306 (PMC12043121; doi:10.1371/journal.pone.0322306)
Supplement: Attachment — Submitted filename: Response_to_Reviewers_auresp_2.docx [file pone.0322306.s017.docx]

**Response to Reviewer’s comments**

We sincerely appreciate the reviewer’s additional comments, which have helped us further improve our manuscript. Below, we provide our response to the reviewer’s comments.

**Minor revisions**

1. Lines 257: -… located on the short arm of chromosome 2A, in close proximity to PPD-A1 (TraesCS2A01G081900; 2A: 36,936,362–36,938,400). The search for 'TraesCS2A01G081900' returned no results. Please recheck and submit these manuscript.

Response: The reason why the gene TraesCS2A01G081900 (2A: 36,936,362–36,938,400) could not be found is that it was annotated in the IWGSC 1.0 assembly version. This information can be verified in Reference 9 of our manuscript. In the IWGSC 2.1 assembly version, the gene has been renamed to TraesCS2A02G081900. Therefore, we have updated our manuscript accordingly, replacing TraesCS2A01G081900 with TraesCS2A02G081900 and specifying the corresponding genomic location.
